# Nutraceutical effects of table green olives: a pilot study with *Nocellara del Belice* olives

**DOI:** 10.1186/s12979-016-0067-y

**Published:** 2016-04-05

**Authors:** Giulia Accardi, Anna Aiello, Valeria Gargano, Caterina Maria Gambino, Santo Caracappa, Sandra Marineo, Gesualdo Vesco, Ciriaco Carru, Angelo Zinellu, Maurizio Zarcone, Calogero Caruso, Giuseppina Candore

**Affiliations:** Sezione di Patologia generale del Dipartimento di Biopatologia e Biotecnologie Mediche (DIBIMED), Università di Palermo, Corso Tukory 211, 90134 Palermo, Italy; Istituto Zooprofilattico Sperimentale della Sicilia, Via Gino Marinuzzi 3, 90129 Palermo, Italy; Dipartimento di Scienze Biomediche, Università di Sassari, Viale San Pietro 43/b, 07100 Sassari, Italy; UOC Epidemiologia Clinica con registro tumori di Palermo e provincia, AOUP “Paolo Giaccone”, Palermo, c/o Dipartimento di Scienze per la promozione della salute e materno infantile “G. D’Alessandro”, Università di Palermo, Via del Vespro 133, 90131 Palermo, Italy

**Keywords:** Table green olives, Mediterranean Diet, Nutraceuticals, Dietary intervention, Oxidative stress, Inflammatory status

## Abstract

**Background:**

The aim of this study was to analyse the nutraceutical properties of table green olives *Nocellara del Belice*, a traditional Mediterranean food. The Mediterranean Diet has as key elements olives and extra virgin olive oil, common to all Mediterranean countries. Olive oil is the main source of fat and can modulate oxidative stress and inflammation, whereas little is known about the role of olives. Moreover, emerging evidences underline the association between gut microbiota and food as the basis of many phenomena that affect health and delay or avoid the onset of some age-related chronic diseases.

**Methods:**

In order to show if table green olives have nutraceutical properties and/or probiotic effect, we performed a nutritional intervention, administering to 25 healthy subjects (mean age 38,3), 12 table green olives/day for 30 days. We carried out anthropometric, biochemical, oxidative stress and cytokines analyses at the beginning of the study and at the end. Moreover, we also collected fecal samples to investigate about the possible variation of concentration of *Lactobacilli*, after the olives consumption.

**Result:**

Our results showed a significant variation of one molecule related to oxidative stress, malondialdehyde, confirming that *Nocellara del Belice* green olives could have an anti-oxidant effect. In addition, the level of interleukin-6 decreased significantly, demonstrating how this food could be able to modulate the inflammatory response. Moreover, it is noteworthy the reduction of fat mass with an increase of muscle mass, suggesting a possible effect on long time assumption of table olives on body mass variation. No statistically significant differences were observed in the amount of *Lactobacilli*, although a trend towards an increased concentration of them at the end of the intervention could be related to the nutraceutical effects of olives.

**Conclusion:**

These preliminary results suggest a possible nutraceutical effect of daily consumption of green table olives *Nocellara del Belice*. To best of our knowledge, this is the first study performed to assess nutraceutical properties of this food. Of course, it is necessary to verify the data in a larger sample of individuals to confirm their role as nutraceuticals.

## Background

Nowadays, ageing process and the related diseases constitute one of the bigger challenges in Western countries. The general increase of lifespan does not go, hand in hand, with the increase of healthy lifespan, the so-called “healthspan”. This constitutes a worldwide problem, in particular due to age-related chronic diseases [1].

It is well known that the pathogenesis of age-related diseases is characterized by a low-grade inflammation. In particular, the visceral adipose tissue is a source of inflammatory mediators produced by adipocytes and infiltrating monocytes [2].

Abdominal obesity with dyslipidaemia, elevated blood pressure and impaired glucose tolerance characterizes metabolic syndrome (MS) that predisposes to the onset of age-related diseases. As many studies demonstrate, a dietary Mediterranean regimen can positively influence these parameters. Large intervention trials showed, in fact, that Mediterranean Diet (MedDiet) could prevent and or delay the onset of age-related diseases with a great implication in the health social system [3–7].

The traditional MedDiet is a common dietary pattern, adopted by inhabitants of countries within Mediterranean basin where the olive tree, *Olea europaea*, is widely cultivated for the production of table olives and oil. They are the essential components of the MedDiet with a very significant economic value. Besides of the economical contribution to national economies, these are important in terms of nutritional value. Extra virgin olive oil (EVOO) has been claimed to play a key role in the prevention of age-related diseases and in the attainment of longevity. This is due to the high levels of monounsaturated fatty acids, likely responsible for the decreased low density lipoprotein levels, and phenolic compounds claimed to play a role as antioxidants and anti-inflammatories [6, 8, 9].

Foods with bioactive molecules can be considered “nutraceuticals”, defined as “Naturally derived bioactive compounds that are found in foods, dietary supplements and herbal products, and have health promoting, disease preventing, or medicinal properties”. The term was coined in 1989 by Stephen De Felice and was born from the conjunction between nutrition and pharmaceutics [10].

As reported in a recent review, table olives are extremely rich sources of polyphenols, especially oleuropein and hydroxytyrosol, comprising 1–3 % of the fresh pulp weight. Despite the high levels of hydroxytyrosol in both table olives and EVOO, in humans its bioavailability was proved only in oil. Accordingly, to the best of our knowledge, there are no human studies on health effects of table olives [11]. However, the amount of polyphenols is strongly influenced by the variety and the geographical origin. *Greek Koroneiki* have a very high level of them, while the polypenol content of the *Spanish Arbequina* is low and that of *Sicilian Nocellara* is medium-high [12]. So, a possible anti-inflammatory and anti-oxidant effect of these Sicilian olives is conceivable.

The development of strategies aimed at counterbalancing the frailty in the elderly is a major challenge for the medicine of 21^st^ century [1]. As recently reviewed, ageing affects the gut microbiota composition and its influence in immune response. Age-related gut microbiota changes are associated with immunosenescence and inflamm-ageing. Hence, the gut ecosystem shows the potential to become a promising target for strategies able to contribute to the health status of elderly. In this context, the consumption of pro/prebiotics may be useful in both prevention and treatment of age-related pathophysiological conditions, favouring the attainment of longevity [13].

Probiotics are defined as “Live microorganisms which when administered in adequate amounts confer a health benefit on the host”. *Lactobacilli* (*L.*) and *Bifidobacteria* are the most commonly used bacterial probiotics [14]. Nutritional supplementation in aged people might help to maintain good immune-inflammatory responses by re-equilibrating the gut microbiota.

Fermentation is one of the oldest methods to preserve olives. It has applied worldwide for thousands of years. The microbiota of olives during fermentation, that varies somewhat from *cultivar* to *cultivar*, has been recently reviewed, showing that *L.* are the major constituents of *Nocellara del Belice* olives microbiota [15, 16]. So, a possible probiotic-like effect of these olives is feasible.

The aim of this pilot study was to evaluate the effect of green table olives *Nocellara del Belice* on clinical and biological parameters of healthy individuals at baseline (T0) and after the assumption of 12 olives/day for 30 days (T1) (this amount was chosen to assure the administration of 2x10^7^*L.*/die, see below).

## Results and discussion

### Hematochemical tests

At the end of the intervention, all hematochemical parameters did not experienced variations, with the exception of alkaline phosphatase that significantly increased (Table 1).

**Table 1:** The Table shows the arithmetic average values at T0 and T1, the p-value and the variation in percentage (+ indicates an increase of the variable at T1; - a decrease at T1)

Variable	T0 ± SD	T1 ± SD	p-value	%
Alkaline phosphate (IU/L)	49.95 ± 13.26	53.73 ± 16.81	0.022	+7.57
Fat mass%	29.70 ± 7.92	28 ± 7.24	0.004	−5.72
Muscle mass %	66.97 ± 7.62	68.36 ± 6.85	0.003	+2.09
IL-6 (FI)	31.52 ± 29.37	20.89 ± 11.93	0.027	−33.73
MDA (μmol/L)	2.72 ± 0.64	2.33 ± 0.49	0.005	−14.24
Weight (Kg)	70.44 ± 14.07	69.93 ± 13.77	0.08	−0.72
BMI (Kg/m^2^)	24.37 ± 4.19	24.24 ± 4.16	0.22	−0.53

*T0* baseline, *T1* the end of the nutritional intervention (30 days), *BMI* Body Mass Index, *IL-6* interleukin-6, *IF* indirect fluorescence, *MDA* malondialdehyde, *SD* standard deviation

However, the increased values were in normal range. This means that a regular consumption of 12 green olives/day for 30 days does not have a detrimental effect on liver and kidney function and on lipid values.

### Anthropometric measurements

At T1, in analysed subjects the fat mass significantly decreased together to an increase of muscle mass (Table 1). The possible explanation could be linked to the capacity of conjugated linoleic acid (CLA) to reduce the body fat levels [17]. This molecule is present both in EVOO and table olives, and can also be produced during their digestion. In experimental models, acting as signalling mediators, CLAs inhibit lipogenesis, increase fat oxidation, and reduce adipocytes size [18, 19].

### Cytokines analyses

The serological analysis of the levels of the main pro and anti-inflammatory cytokines was conducted. Although it was not possible to evaluate the absolute concentration of interleukin (IL)-6 because it is too low, a significant variation was measured in the indirect fluorescence (IF). In fact, its levels significantly decreased at the end of the dietary intervention (Table 1).

IL-6 is a pleiotropic cytokine capable of regulating proliferation, differentiation and activity in a variety of cell types. In particular, it plays a pivotal role in acute phase responses and in the balancing of the pro and anti-inflammatory pathways. It is involved in impaired lipid metabolism and in the production of triglycerides. Moreover, it decreases lipoprotein lipase activity and monomeric lipoprotein lipase levels in plasma which contributes to increased macrophage uptake of lipids [20]. This datum suggests that a regular consumption of green olives can have anti-inflammatory effects linked to polyphenols, known to have anti-inflammatory properties [6].

### Oxidative stress analyses

At the end of intervention, the values of malondialdehyde (MDA) significantly decreased (Table 1), while paraoxonase (PON) plasma levels and reduced glutathione in the red blood cells were not changed (data not shown). MDA is the main product of the polyunsaturated fatty acids peroxidation and is an important index of oxidative stress [21]. So, its reduction should be linked to the increased assumption of mono-unsatured oleic acid by olives.

### Microbiological analyses on feces

The amount of *L.*/g of feces was quantified before and after the intervention. No statistically significant differences were observed, although a trend towards an increased amount of *L.* was highlighted in some subjects at T1 (data not shown). Thus, we can speculate that a more durable dietary intervention and a bigger sample of people could give more interesting results.

## Conclusions

The traditional MedDiet is a common dietary pattern that identify a lifestyle and a culture. It was proven that it contributes to better health and quality of life. Concerning its healthy effects, low content of animal protein and low glycaemic index may directly modulate the insulin/insulin-like growth factor-1 and the mammalian target of rapamycin pathways, known to be involved in ageing, age-related diseases and longevity. In addition to the influence on nutrient sensing pathways, many single components of MedDiet are known to have positive effects on health, reducing inflammation, oxidative stress and other important risk factors of age-related diseases [6].

This pilot study demonstrates an anti-inflammatory and anti-oxidant effect of daily consumption of green table olives *Nocellara del Belice*. Moreover, it is noteworthy the reduction of fat mass with an increase of muscle mass. Although no statistically significant probiotic effect was observed, the positive trend related to *L.* amount at T1 could represent a starting point for further studies.

It is to note that the study presents limitations. One is strictly related to the intrinsic complexity of human as study model and to the inter/intra-individual variability. These features are more evident in ageing than in younger people. This is the reason why we chose middle age people. So, our choice represented the second limitation of the study because we did not analyze the effects of the intervention in elderly. Thirdly, it is necessary to verify these data in a larger sample of individuals to confirm the role of table green olives as nutraceutical foods. Also the duration of the intervention could be inadequate. In fact, we developed a short-term dietary regimen (30 days). This is a good choice in terms of compliance to the study because the more is the time of intervention the more is the drop out effect. But, a long-term dietary intervention could be stronger in terms of variation of analyzed parameters *(*e.g.*, L.* amount in feces).

However, these new knowledges give an important achievement for the food and farming industry, especially in Sicily, where the olives represent a great potential resource. No approved healthy property and claim exist for them. Therefore, adding such a common product to the class of nutraceuticals could represent a big deal.

In the era of many expansive and mysterious longevity elixirs, the olives could represent a traditional, cheap and accessible to everyone “healthy food”.

## Methods

### Study design

The trial consisted in the assumption of 12 olives/day for 30 days. They belonging to the variety *Nocellara del Belice*, were processed in salt solution without any chemical additives.

**Fig. 1:**
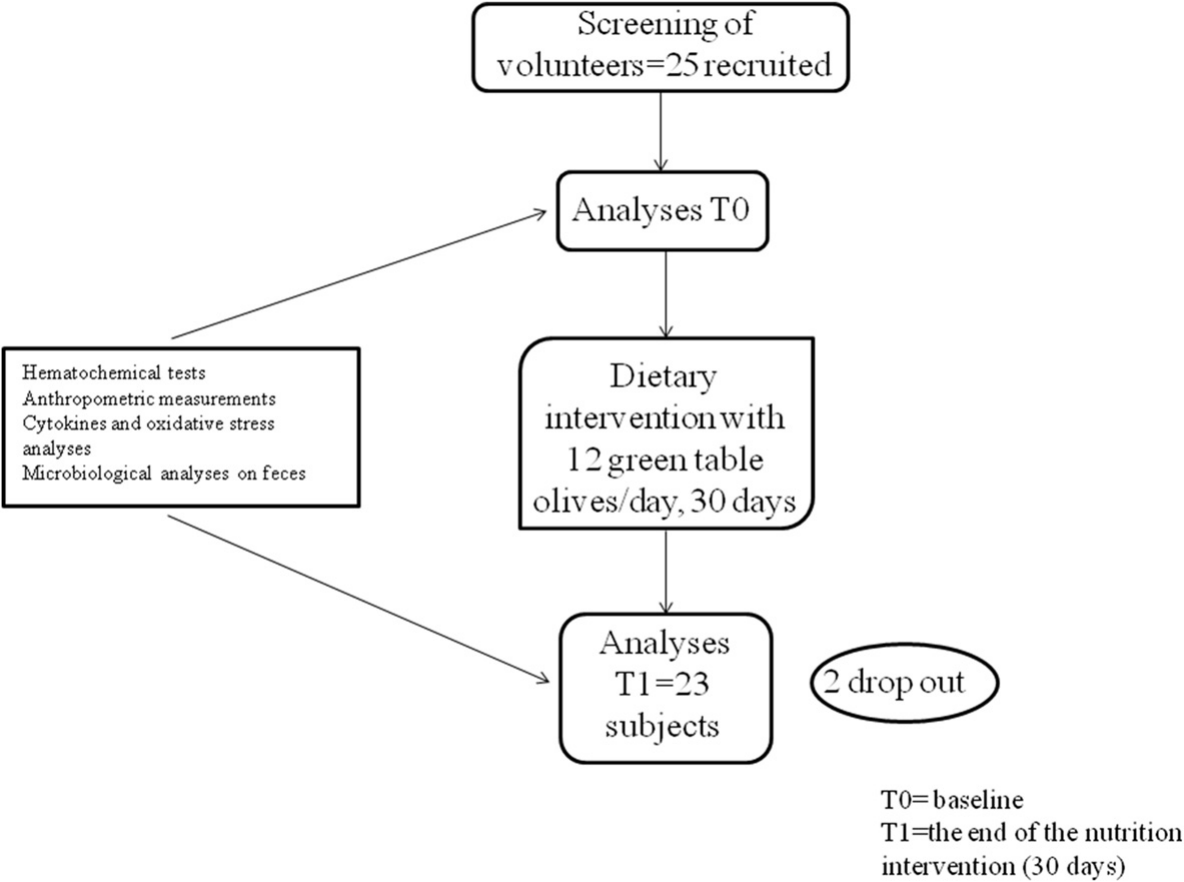
The figure shows the flow chart of the study design

See Fig. 1 for the flow chart of the study design.

### Study population

Twenty-five randomized volunteers (mean age 38,3), both men and women, were recruited from April 2015 to July 2015. The subjects included were: healthy, with age between 18 and 65 years and Caucasian. The exclusion criteria provided: a history of the absence of pathologies (obesity, MS); a history of use of any pre or probiotics as dietary supplements within 3 months prior to the study; a history of treatment with statins or similar and with lyposoluble drugs; the onset of gastrointestinal disorders or the use of antibiotics during the nutritional intervention. No restriction related to sex was considered. Two subjects dropped out of the trial. All participants signed an informed consent before the enrolment. To respect the privacy, everyone was identified with an alphanumeric code. Height and weight were measured wearing light clothes and barefoot. The body composition was registered using specific hardware and software. Body mass index was calculated as weight (in kilos) over height squared (in square metre) (Table 1). Dietary habits were assessed through a food frequency questionnaire, officially validate by the EPIC study. Blood tests, oxidative stress and cytokines analyses were carried out for all subjects at T0 and T1. Molecular analyses were conducted on *L.* DNA obtained from fecal samples to measure the variation of its amount. A database was created to insert all participants’ data and to handle the collected information.

### Hematochemical tests

The recruited people underwent to venipuncture at T0 and at T1. Blood samples were collected in specific blood collection tubes containing ethylenediaminetetraacetic acid (EDTA) for plasma analyses and in serum tubes with no additives. Plasma and sera were separated from whole blood by low-speed centrifugation at 2,500 rpm for 15′ at 4 °C. After separation, the samples were stored at −80 °C for further tests. We also obtained gruel of red blood cells from blood collected in tubes with EDTA through three washes with physiological solution (centrifugation at 2,500 rpm for 15′ at 4 °C).

### Evaluation of parameters of oxidative stress

These analyses were conducted in collaboration with the University of Sassari. Thiobarbituric acid reactive substances (TBARS) were determined according to the method described by Esterbauer and Cheeseman [22]. TBARS methodology measures MDA and other aldehydes produced by lipid peroxidation induced by hydroxyl free radicals. For the measurements, plasma was mixed with 10 % trichloroacetic acid and 0.67 % thiobarbituric acid and heated at 95 °C in thermoblock heater for 25′. TBARS were determined by measuring the absorbance at 535 nm. A calibration curve was obtained using standard MDA and each curve point was subjected to the same treatment as that of the samples.

PON activity was determined by measuring the increase in absorbance at 412 nm (formation of 4-nitrophenol), using paraoxon (O, O diethyl-O-p-nitrophenyl phosphate) as a substrate [22]. The enzyme activity was calculated by using the molar extinction coefficient of 17,100 M^−1^cm^−1^ and one unit of PON activity was defined as 1 nanomole of 4-nitrophenol formed per minute. For red blood cell glutathione quantification, 200 μl (μL) of thawed packed cells were lysed by adding 600 μL of cold water and keeping the samples at 4 °C for 15′. 200 μL of lysed samples were deproteinized by adding 200 μL acetonitrile and centrifuged at 2,000 × g for 5′. Samples were then derivatized by mixing 100 μL of supernatant with 100 μL of sodium phosphate buffer (60 μmol (mmol)/L, pH 12.5), and 25 μL of 5-Iodoacetamidofluorescein (4.1 mmol/L). After vortex mixing, samples were incubated for 15′ at room temperature. Derivatized samples were diluted 100-fold in water and analysed by capillary electrophoresis with laser induced fluorescence detection [23].

### Pro and anti-inflammatory cytokines analyses

These analyses were conducted using Luminex assays, coupled to Bio-Plex Manager software.

Data obtained have been checked by technical department and quality control parameters. Values of the standard curve were compared to the values provided by the manufacturer of the kits used and must not exceed a CV of 15 %. All of above parameters were applied on, at least, the 90 % of the standard curve values.

### Microbiological and molecular analyses of *Lactobacilli*

In order to quantify the amount of *L.* in each olive, 1 g of pulp was suspended in phosphate-buffered saline solution (1 mL), homogenized for 2′ at maximum speed, and then serially diluted. Decimal dilutions were plated and incubated on *de Man, Rogosa and Sharp*e at 30 °C for 48 h to observe the *L.* growth. The colonies’ count was performed in triplicate and the *L.* DNA was extracted from them to perform molecular analyses. Moroever, colony suspension were used as a template for Real Time PCR. The primers and probes used to detect *L. species* (*spp*) were based on 16S rRNA gene sequences retrieved from the NCBI databases (Table 2). The amplification reactions were carried out in a total volume of 25 ml containing 1X SSoFast Probe mix (BIORAD), primers (each at 200 nM concentration), 100 nM TaqMan MGB probe, 60 ng purified target DNA. Amplification (1 cycle of 5′ at 95 °C, 45 cycles of 15″ at 95 °C and 1 cycle of 1′ at 60 °C) and detection were carried out on a CFX Real Time system (BIORAD).

**Table 2:** Primer and probe sequences for analyses of *L. spp* in Real Time PCR

	Oligonucleotide sequence 5′-3′
Primer Forward	GAGGCAGCAGTAGGGAATCTTC
Primer Reverse	GGCCAGTTACTACCTCTATCCTTCTTC
Probe	FAM-ATGGAGCAACGCCGC-QUENCER

Fluorescent probe was labeled at 5′ end with the reporter dye 6-carboxyfluorescein and at 3′ end with a quencher dye. A negative and a positive control were included on the reaction plate.

In order to perform the quantification of *L.* in each feces sample, the QIAamp DNA Stool Minikit (Qiagen) was used to extract DNA from an appropriate amount of frozen stool sample, according to the manufacturer’s instructions. The Real Time PCR was performed as previous described and the cycle threshold of each sample was compared to a standard curve made by diluting genomic DNA (10-fold serial dilution) from cultures of known concentrations of *L.* (10^6^ CFU/ml).

### Statistical analyses

The paired comparisons were performed with the Student’s *t*-test or the Wilcoxon signed rank test, according to the normality of samples. Statistical analyses were performed with the IDE RStudio for the R (version 3.2.2) software [24, 25].
